# Late-Onset Endophthalmitis Secondary to Exposed Glaucoma Tube Implant in a Rare Case of Paediatric Glaucoma

**DOI:** 10.1155/2011/183647

**Published:** 2011-12-17

**Authors:** Akshatha Ranganath, Adnan Hashim

**Affiliations:** Department of Ophthalmology, Rotherham District General Hospital, Rotherham, South Yorkshire S60 2UD, UK

## Abstract

Glaucoma drainage implants (GDIs) are used to treat paediatric glaucoma resistant to conventional medical and surgical treatment, achieving good intraocular pressure (IOP) control and long-term success. Late endophthalmitis is a rare complication that may develop following GDI surgery. A 17-year-old male presented with acute endophthalmitis 2 years after Ahmed glaucoma valve implantation with pericardial patch graft for management of refractory glaucoma secondary to congenital ectropion uveae. The glaucoma tube was exposed due to erosion of the overlying conjunctiva with no visible pericardial graft. After control of active infection, he underwent tube revision surgery whereby the exposed tube was retained and repatched with a double-thickness pericardial patch graft. He did well following surgery with good control of IOP and restoration of vision. Conjunctival dehiscence with graft melting over the GDI tube presented a major risk factor for endophthalmitis. Prompt surgical revision of an exposed tube is highly recommended to avoid ocular morbidity.

## 1. Introduction

We present a rare case of delayed-onset acute infective endophthalmitis in a young male secondary to erosion of exposed glaucoma drainage tube in an intractable developmental glaucoma due to congenital ectropion uveae (CEU). It highlights many important clinical points like the high incidence of glaucoma in CEU, difficulties in achieving glaucoma control with conventional medical treatment, and the challenges and complications encountered with surgical management.

## 2. Case Report

A 17-year-old Caucasian male presented with gradual onset of right eye pain, redness, photophobia, blurred vision, and severe headache progressing gradually over 5 days. He was started on chloramphenicol eye drops by his general practitioner that he used for 3 days with no improvement of symptoms.

**Figure 1:**
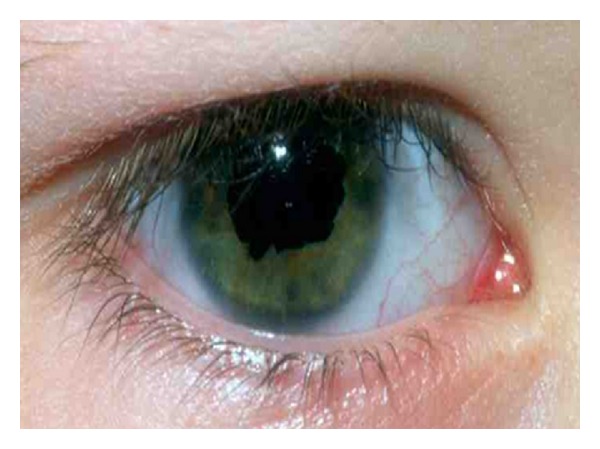
Right eye at 4 years of age showing congenital ectropion uvea 360° around the pupil extending partially and irregularly onto the iris surface.

He was diagnosed with right anterior segment dysgenesis in the form of congenital ectropion uveae at 4 years of age (Figure 1). He was on maximal medical therapy to control IOP with no success. Therefore, he underwent right glaucoma tube implant (Ahmed Glaucoma Valve) surgery with 0.2 mg/mL of Mitomycin C and pericardial patch graft in June 2009 at 15 years of age. Following surgery, he was on timolol maleate 0.5% eye drops once a day to the right eye. He was otherwise healthy with no other significant pastmedical or family history.

**Figure 2:**
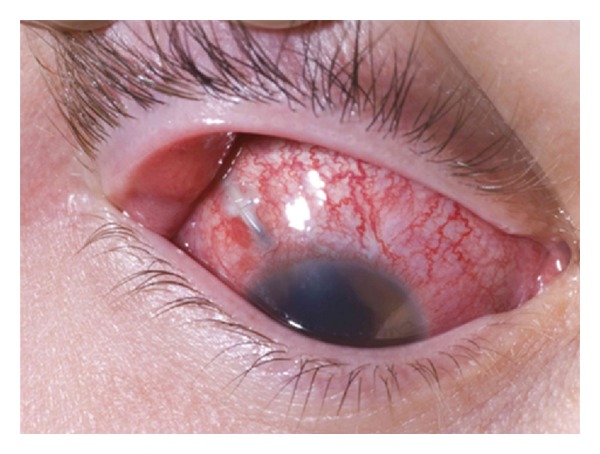
Right eye: exposed glaucoma tube implant in the superotemporal quadrant due to erosion of the overlying conjunctiva with no visible pericardial patch graft.

**Figure 3:**
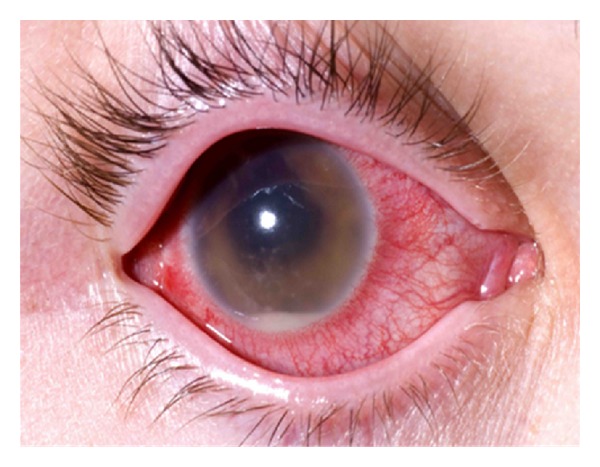
External photo of right eye showing an inflamed conjunctiva, hazy cornea, and hypopyon in the anterior chamber.

On ocular examination, vision in right eye was hand movements and left eye was 6/6. The right eyelids were swollen and inflamed. The conjunctiva was injected and chemosed with a 5 mm conjunctival dehiscence directly over the tube in the superotemporal quadrant along with tube exposure 4 mm posterior to the limbus with a loose suture overlying the tube (Figure 2). There was corneal oedema with fresh keratic precipitates on the corneal endothelium. The anterior chamber was well formed with presence of hypopyon, and 2.5 mm of well-positioned tube was visible in the superotemporal quadrant (Figure 3). The iris showed prominent ectropion uvea surrounding the entire pupillary border and extending about 2 mm onto the iris surface. Pupillary reaction was sluggish with posterior synechiae at six “0”clock position and IOP of 28 mmHg. After multiple instillation of dilating drops, the pupil dilated to 4-5 mm, and posterior synechiae was broken. Posterior segment examination of right eye showed a clear lens with presence of severe vitritis and no fundal view. The left eye was completely normal with IOP of 12 mmHg, and fundus showed a pink disc with a cup-disc ratio of 0.2, healthy vessels, and macula.

Ultrasound scan of the right eye confirmed vitreous haze with a flat retina. A diagnostic vitreous tap was performed, and the specimens were directly smeared for Gram stain and plated for culture and sensitivity. (The result was negative despite strong clinical evidence of infective endophthalmitis). A diagnosis of right early endophthalmitis secondary to exposed glaucoma tube implant was made.

The patient received intravitreal injection of Vancomycin (1 mg in 0.1 mL) and Amikacin (0.4 mg in 0.1 mL) and was started on topical antibiotics—Ofloxacin hourly, Prednisolone acetate 1% every 4 hours, and Cyclopentolate 1% TDS. After control of active infection in the right eye, he underwent tube revision surgery at a tertiary care eye unit. The exposed tube was retained and repatched with a double-thickness pericardial patch graft. Due to the lack of spare conjunctiva to cover the graft, a bandage contact lens was placed on the eye for mechanical protection and to promote healing. The patient did well following surgery with good control of IOP. At 3-month followup his vision in the right eye had improved to 6/12, and IOP was 16 mmHg with no other antiglaucoma medications. Fundus examination of right eye showed long-standing glaucomatous changes of the optic nerve with cup-disc ratio of 0.9.

## 3. Discussion

Paediatric Glaucoma is a relatively rare, potentially blinding condition characterised by elevated IOP. Overall, glaucoma is responsible for about 5% of blindness in children worldwide [1]. It is classified as primary when an isolated idiopathic developmental abnormality of the anterior chamber angle exists and secondary when aqueous outflow is reduced due to either a congenital or acquired ocular disease [2]. Secondary paediatric glaucoma is commonly associated with anterior segment dysgenesis (ASD), developing in 50% of cases [3].

Congenital ectropion uveae is a rare form of ASD that may be present in one or both the eyes, consisting of iris pigment epithelium on the anterior surface of the iris, anterior insertion of the iris, dysgenesis of the drainage angle, and glaucoma [4]. It may be associated with other ocular and systemic anomalies like neurofibromatosis, Rieger's anomaly, and Prader-Willi syndrome. However, not all cases have systemic associations [5]. Although the actual anomaly is nonprogressive, multiple studies have linked it with the appearance of progressive open-angle glaucoma at birth, infancy, or later in life secondary to angle dysgenesis. IOP shows an initial decrease when medication is administered but rises again soon after often leading to intractable glaucoma. Surgery is the definitive treatment modality for the control of IOP [6].

Trabeculectomy, which is most often chosen, has met with limited success in paediatric glaucoma patients, and cycloablative therapy has a relatively high rate of complications and frequently necessitates retreatment in children [7].

The Ahmed glaucoma valve (AGV) has good success rate in controlling IOP in paediatric glaucoma as proven by various studies [7, 8]. A patch graft placed over the silicone portion of the tube is a simple preventive measure that helps to protect the conjunctiva from erosion. Currently used grafts are prepared from a variety of materials such as sclera, fascia lata, and different types of pericardium. The reported advantages with pericardial patch grafts include uniform size and quality, commercial availability without dependence on an eye bank, potentially lower costs, and a processing method that leads to enhanced immunologic safety and reduced risk of viral transmission. However, melting of graft materials resulting in tube exposure is a recognised complication after use of any donor patch material [9–11].

Conjunctival erosion and tube exposure are significant risk factors for the development of endophthalmitis in eyes with glaucoma drainage devices [12]. It can result from mechanical abrasion of the conjunctiva over the tube by the eyelid, excessive conjunctival tension over the tube, tube mal-position, or lack of a smooth and tapered surface between the patch graft and host. In addition to this eye rubbing and poor ocular lubrication could increase the possibility of conjunctival erosion [11]. The risk of endophthalmitis is about five times higher following AGV implant surgery in the paediatric age group as reported by Al-Torbak et al. [13]. The eroded conjunctiva surrounding the tube probably serves as a conduit by which normal flora may pass from the ocular surface into the eye.

Prompt repair of tube erosion is imperative in order to resolve the exposure with good tissue coverage while avoiding interference with the position or function of the implant and minimising the likelihood of future tube re-exposure. In our patient as the IOP was well controlled with the AGV it was worth trying to preserve the tube after clearing the infection. However, the fragile conjunctiva can be difficult to manage, especially in the setting of recent infection. Simple conjunctival closure without a patch graft is not recommended, as conjunctiva does not reliably remain closed over immediately underlying synthetic materials such as plastics, silastic, silicone, or polypropylene. A patch graft of collagenous human tissue is necessary to prepare the eye for complete healing and resolution of the exposure. Double-thickness pericardium patch grafts are less likely to be associated with conjunctival erosion and tube exposure than single-thickness pericardium patch graft [14]. The management of endophthalmitis from an exposed glaucoma drainage tube is extremely challenging due to the high risk of further infection, recurrent tube erosion, and exposure.

In conclusion, we would like to stress the importance of long-term and regular followup of patients especially children with glaucoma drainage devices in order to identify tube erosion at an early stage and correct it. Also, these patients need to be educated to avoid rubbing their eyes and to seek medical help as soon as they develop any symptoms of eye infection to avoid ocular morbidity.
